# Two Fatal Cases of Landry's Paralysis

**Published:** 1909-09-11

**Authors:** 


### MEDICINE.

### TWO FATAL CASES OF LANDRY'S PARALYSIS.

LANDRY'S paralysis—acute ascending paralysis—is not common; yet it is not so rare but that most practitioners come across it sooner or later. The following account of two recent cases may, therefore, be of interest:—

A man, aged 57, a journalist, came under observation on July 28. He had previously enjoyed excellent health, was neither alcoholic nor syphilitic, and came of a healthy and long-lived family. On July 17, when getting up in the morning, he felt a curious sensation in his left foot with a sense of slight loss of power in it. The feeling of weakness spread steadily upwards to the calf and knee. Then, during the day, tingling sensations and weakness developed in the tips of the fingers of the left hand, spreading thence to the hand and arm a little later. The right upper limb also seemed to be affected, though the abnormal feeling was less marked here than on the left side. Next day, July 18, the patient was quite unable to rise. Both lower limbs were helpless, and so was the left arm. By July 19 all four limbs were completely paralysed.

When seen on July 28 the man was found to be very corpulent; he was lying on his back in bed, incapable of moving either limb or trunk. He could still move his head easily; speech and brain power were good; there were no difficulties in breathing or swallowing. Percussion of the paralysed muscles evoked local fibrillary contractions. Excitability to the faradic current was much diminished, without there being any notable increase in excitability to galvanism. The cremasteric reflexes were abolished. The pharyngeal and the pupillary reflexes were normal. There was no headache, nor was any pain complained of. The sphincters were natural. There were no visceral symptoms, no albuminuria, no coldness nor cyanosis of the extremities.

Some days after admission to hospital the toes began to exhibit contractions. Little by little the patient succeeded in making very limited movements of his feet, of his fingers, and of his shoulders.

On August 19 diarrhœa set in. There were six motions in the night. Next day this diarrhœa persisted, though the general condition remained good. On August 21 the patient was seized with dyspnœa at 3 A.M., this symptom becoming rapidly and progressively worse until death took place at 7 A.M., without any agitation or convulsions.

At the post-mortem examination the lungs were very congested and voluminous. The whole body was livid, and so were all the viscera. The heart and aorta were healthy. The brain exhibited lividity of its meninges, but no gross lesion of cortical, central, cerebellar, or bulbar parts. The cerebral arteries were sound. Histological examination of the spinal cord and peripheral nerves exhibited no obvious lesion, except slight neuritis of the sciatic nerve and of the nerves of the forearm. The vagi exhibited no lesion at all.

The second was a man aged 35 when admitted to hospital on October 28. He was a railway guard. He had been in perfect health until one morning, 25 days before he came under observation, when he noticed a certain amount of disability when he moved his head. At first he paid little attention to it, thinking it was an ordinary stiff neck. During the day, however, the trouble increased, and spread from his neck to his limbs. He found it difficult to walk along the platforms when his train stopped at stations, and it became impossible for him to help in getting the luggage in and out of his van. He went to bed without doubting he would be better after a night's rest; but next day he was worse, and he sought medical advice. At first the doctor regarded the symptoms as rheumatic, saying that they were not serious. The patient dragged himself home, took to his bed at once, and never left it afterwards. Paralysis rapidly became complete.

When seen in hospital on October 28 he was a big man, very stout, lying helpless on his back. His extremities were rather cold and decidedly livid. Intelligence was perfectly retained; the history he gave was clear and concise. The upper and lower limbs were absolutely inert. Neither arm and neither leg could be moved upon the plane of the bed, still less raised above it. The trunk was equally paralysed. When the man was asked to try and sit up not a single muscle could be detected moving. The muscles of the neck and face, on the other hand, were natural; the head could be turned to either side at will. There was no ocular paralysis. Swallowing was imperfectly performed, fluids particularly often finding their way into the larynx and causing a troublesome cough, which made the lividity still worse. There was no pain; the main discomfort was in regard to breathing. Sensations were unimpaired. Both knee-jerks were abolished; the plantar reflexes were flexor. The sphincters were not paralysed. There were no trophic changes, no wasting of muscles. The cyanosis and the coldness of the extremities seemed to be due to the impairment of respiratory movements. The patient looked like one being suffocated. On auscultation a few subcrepitant râles could be heard at the bases of the lungs. The heart was beating faster than normal (130), but otherwise it seemed natural. The appetite was good, the tongue clean and moist. The urine contained neither sugar nor albumin.

On October 30 the paralysis was neither better nor worse, but the dyspnœa was notably greater. The skin generally was cyanosed, the face purplish, the eyes injected. The patient, unable to move his body, was constantly moving his head about as though seeking for more air. He kept on asking nurse to remove the mucus which re-accumulated in his throat, and which he could not get rid of for himself because he was quite unable to cough. Oxygen inhalations and syrup of ether were the only things that gave him any relief. Half an hour later he fell asleep, and two hours after this he suddenly ceased to breathe, without any struggling and without waking up.

At the post-mortem examination the lungs were engorged with blood, but not œdematous. The heart weighed a little more than normal, but it looked healthy; the aorta was free from atheroma. The liver, kidneys, spleen, suprarenal capsules, pancreas, stomach, and intestines were all normal, except for lividity. The brain was congested, but exhibited no obvious lesion; its arteries and meninges were healthy.

Histologically, except for much passive congestion, the lungs, liver, and kidneys presented no disease. The myocardium was natural. The spinal cord and the medulla oblongata showed no definite abnormality either, nor did the peripheral nerves, nor the muscles that had been paralysed.

Cerebro-spinal fluid had been obtained by lumbar puncture during life; it exhibited a few red and white corpuscles, but only in the same proportions as in the blood; there was no meningitic reaction.

In these two patients the symptoms were almost exclusively motor, which is one of the marked features in Landry's disease. The paralysis is widespread and progressive. In the first patient the onset was in the left leg, spread thence to the arm of the same side, then to the other arm, and lastly to the right leg; it was, it will be noted, ascending on the one side, but descending on the other. In the second case the first symptoms appeared in the muscles of the neck, then were noticed in the legs, and lastly in the arms. These irregularities in the direction of spread—departures from the strictly "ascending" course—are so common as to be the rule rather than the exception in these cases. There is even a strictly descending type, which is the probable diagnosis in the case of the celebrated naturalist, Cuvier. In most cases there is, to start with, a simple paresis, which gradually but steadily spreads until it finally becomes complete; but the order of onset and of spread is by no means constant.

Cases that end fatally do so, as a rule, by interference with breathing; this may sometimes be due to paralysis of the intercostal muscles followed by paralysis of the diaphragm, suggesting changes in the cervical enlargement of the cord. In other cases the coincident tachycardia suggests a lesion in the medulla oblongata. It is often stated that it is unusual for the fatal respiratory failure to occur if the patient has already survived the third week, but both the above cases are exceptions to this so-called rule—one died on the twenty-seventh day, the other on the thirty-fifth; and in the latter hopes of recovery had already been aroused because some slight degree of voluntary movement had begun to return. This teaches us that we must be in no great haste to give a favourable prognosis, even when the third week has gone by.

Dyspnœa in Landry's paralysis is not always either sudden or immediately fatal. In the first of the above cases it is true that death occurred in three hours from its onset, but in the second dyspnœa was already well-marked when the man was seen on the twenty-fifth day, and it persisted and increased for two days more before death occurred.

Finally, it is interesting to note that post-mortem examination in the above two patients, as in all cases of Landry's paralysis hitherto described, revealed no adequate cause of death. This may be due to the acuteness of the disease, affecting the nerve tissue but not allowing it time to exhibit obvious pathological changes. Acute death of a tissue without departure from normal structure is well seen in bone in cases of acute suppurative osteitis. On the other hand, the disease may be one which paralyses motor end-plates and produces no changes in the rest of the neuro-muscular chain—comparable to curare poisoning, for instance. In any case, the fact remains that Landry's paralysis is a cause of death of which the post-mortem evidence is almost nil—an argument against the dissociation of clinical and pathological evidence in coroners' courts.

